# Apremilast Add-On Benefits Over Conventional Drugs (ABCD) in Unstable Non-segmental Vitiligo: A 12-Week Single-Center Randomized Controlled Trial

**DOI:** 10.7759/cureus.37180

**Published:** 2023-04-05

**Authors:** Sakshi Sharma, Abhishek Bhardwaj, Pradeep Dwivedi, Suraj Singh Yadav, Muhammad Aaqib Shamim, Surjit Singh, Prem Prakash Sharma, Sneha Ambwani, Kuldeep SIngh

**Affiliations:** 1 Pharmacology, All India Institute of Medical Sciences, Jodhpur, IND; 2 Dermatology, All India Institute of Medical Sciences, Jodhpur, IND; 3 Pediatrics, All India Institute of Medical Sciences, Jodhpur, IND; 4 Community and Family Medicine, All India Institute of Medical Sciences, Jodhpur, IND

**Keywords:** autoimmune diseases, dlqi, bsa, vasi, phosphodiesterase 4 inhibitors, apremilast, unstable vitiligo, non-segmental vitiligo

## Abstract

Background

Apremilast is an oral phosphodiesterase-4 enzyme inhibitor that modulates the immune system by increasing intracellular cyclic adenosine monophosphate levels and inhibiting inflammatory cytokines synthesis. We aimed to compare the efficacy and safety of add-on apremilast in combination therapy with standard treatment in patients with unstable, non-segmental vitiligo.

Methods

The study was a 12-week randomized, controlled, parallel-group, open-labeled trial. The control group received standard treatment (n=15), and the intervention group received 30 mg apremilast twice daily in addition to standard treatment (n= 16). Time to the first sign of re-pigmentation, halt in progression, and change in vitiligo area scoring index (VASI) score is the primary outcomes. Normality was assessed, and appropriate parametric and nonparametric tests were undertaken.

Results

Thirty-seven participants were randomized into two groups, and analysis was done on thirty-one participants. Over the treatment duration of 12 weeks, the median time to observe the first sign of re-pigmentation was four weeks in the add-on apremilast group compared to seven weeks in the control group (p=0.018). The halt in progression was observed more in the add-on Apremilast group (93.75%) compared to the control group (66.66%) (p=0.08). The VASI score decreased by 1.24 in the add-on apremilast group and 0.05 in the control group (p= 0.754). Parameters including body surface area, dermatology life quality index, and body mass index reduced significantly, while the visual analog scale increased significantly in the add-on apremilast group. However, results were comparable between groups.

Conclusions

Treatment with add-on apremilast accelerated clinical improvement. It also reduced disease progression and improved the disease index among participants. However, add-on apremilast had a lower tolerability profile than the control group.

## Introduction

Vitiligo is an acquired chronic disorder of the skin with melanocyte destruction and the loss of pigment melanin which provides color to our skin, mucosa, and hair. Therefore, resulting in typical non-scaly chalky white macules [1]. The specific pathology is not known but may be related to genetic, immunological, and neurological factors [2]. Multiple mechanisms involving autoimmunity, cytotoxicity, oxidation-antioxidation, and neural factors have been implicated [3,4]. Vitiligo affects nearly 1%-2% people globally, with the highest incidence recorded in the Indian subcontinent, followed by Mexico and Japan [5]. The incidence of vitiligo is 0.25%-2.5% in India [6]. The states of Gujarat and Rajasthan have the highest prevalence, around 8.8% [7]. The most common form of the disease is non-segmental vitiligo (accounts for 85% to 90% of cases overall). Still, segmental vitiligo may account for 30% of childhood cases because of its earlier onset [8]. Both genders are equally affected, having no apparent differences in the rate of occurrence as per skin type or race.

Current management includes topical and systemic glucocorticoids, topical calcineurin inhibitors, antioxidants, and narrowband ultraviolet B phototherapy used to promote re-pigmentation; they all have varying degrees of success and adverse effects associated [9,10]. Hence, alternative and more efficacious therapies with better safety profiles for prolonged use are needed to treat vitiligo. Apremilast is an oral medication. It selectively inhibits the phosphodiesterase-4 enzyme and is involved in immunomodulation via elevation of intracellular cyclic adenosine monophosphate (cAMP) and inhibition of Interleukin (IL) - 2 and 8, interferon (IFN) - γ and tumor necrosis factor (TNF) production [11]. It is approved by the Food and Drug Administration (FDA) for treating psoriasis, psoriatic arthritis, and oral ulcers of Bechet’s disease [12-14]. Therefore, based on previous efficacy and safety data, we have conducted this randomized controlled trial to assess the safety and efficacy of apremilast in vitiligo among patients from Rajasthan state of India. The primary objective of the study was to estimate the change in the proportion of responders to add-on apremilast from baseline to week 12, the efficacy of apremilast at the label dosage of 30 mg twice daily with proper titration, in combination therapy with standard treatment versus standard treatment only for re-pigmentation in patients with unstable, non-segmental vitiligo.

## Materials and methods

Study design, sample size, and ethical aspects

The study was a prospective, open-label, parallel-group, randomized controlled trial of 12-week treatment duration, conducted at the outpatient dermatology department of a tertiary care hospital in western India from July 2019 to March 2021. It was a proof of concept and pilot-level study in which sample size was calculated using a two-tailed significance level of 0.05 and 80% power. The effect size was determined using Cohen’s predetermined criteria [15]. With an effect size of 0.8, the required sample size is 25 in each group (total sample size of 50). Due to COVID-19 restrictions, we could only recruit 37 patients for the study. All procedures followed were by the ethical standards of the responsible committee on human experimentation and with the Helsinki Declaration and National Ethical Guidelines for Biomedical and Health Research involving Human Participants, 2017 of Indian Council Medical Research (ICMR). The study was approved by the Institutional ethics committee (AIIMS/IEC/2019-20/848) and is registered with Clinical Trials Registry, India (ICMR-NIMS) (CTRI/2019/07/020234). 

Selection, enrolment of patients, and data collection

The patients with unstable non-segmental vitiligo (diagnosed by a dermatologist) aged 18-60 years willing to participate in the study were recruited after taking written informed consent. Unstable lesions are the lesions that have not been same over six months and are continuously progressing or varying in shape and size. There may be occurrence of new patch/progression of existing patch/presence of the Koebner phenomenon in cases with vitiligo for six months and who were in general good health based on medical history, and physical examination without any serious comorbid condition, were recruited. Patients with any clinically major illness, segmental or mixed vitiligo, acrofacial vitiligo only, pregnant or breastfeeding women, any known allergy to apremilast in the past, and patients on any drug with pigmentation potential were excluded from the study. The investigators evaluated, and pictures were taken for future reference whenever possible. As the data collection was conducted during COVID-19, electronic media like photographs and video calling platforms were the primary modes of data collection, and various scores were evaluated. Subjective variations were tried to be minimized, and a single investigator evaluated all the scores in every patient. Still, due to variations in picture quality of photographs sent by the patients, some variations might be possible in evaluation.

Interventions and study group allocation

The enrolled patients of unstable non-segmental vitiligo were randomized 1:1 to the add-on apremilast (30 mg orally twice daily) intervention group and the standard treatment control group. The computer-generated random table was used for the simple randomization of study participants. The allocation sequence was generated by a person independent of the study. The allocation concealment was performed using serially numbered opaque envelopes. The dermatologist assigned participants to intervention and control groups. However, it was an open-label study. In the intervention group, apremilast was given with a titration pack at the initiation of the study and afterward at the dose of 30 mg twice a day, along with the standard treatment prescribed by consulting dermatologist. In contrast, the control group received standard treatment only. Standard treatment contained physician preferred medicine cocktails depending on the extent of vitiligo and the body parts involved. Standard therapy included a range of treatment strategies (number of patients received therapy among standard treatment group/among add-on apremilast group) such as NB-UVB (2/2), oral steroids (4/8), topical steroids (17/19), azathioprine (0/1), tacrolimus (15/17), melanocyl lotion (2/2), antioxidants (6/2), calcium (2/6), sper lotion (1/2), and patchex tablet (2/1).

Each group was followed up after two weeks, four weeks, and then monthly after two and three months. The flow of participants through each stage of a randomized trial is represented in accordance with the recommendation of CONSORT (Consolidated Standards of Reporting Trials). 

Clinical variables for analysis

The primary outcomes of the study were proportions of patients with halt in progression, with repigmentation, and time to first sign of repigmentation from baseline to week 12 in the add-on apremilast group compared to standard care. The secondary outcomes were to investigate changes in Body Surface Area (BSA), VASI (Vitiligo Area and Severity Index) score, Dermatology Life Quality Index (DLQI) score, and Visual Analogue Scale (VAS) score of vitiligo at week 12 compared to baseline and also to document the number of Adverse Events in 12 weeks. The inter-group comparison of time to the first sign of repigmentation was analyzed using an independent t-test. The relationship between median change from baseline was analyzed using nonparametric tests (Wilcoxon signed ranks test & Mann Whitney U test for paired and unpaired comparison) as the baseline data were skewed and included a few outliers. For non-parametric data, the Friedman test with post hoc Wilcoxon signed rank test on each combination was conducted. Both the groups were individually analyzed for the effect of the given treatment. A p-value of <0.05 was considered statistically significant.

## Results

**Figure 1:**
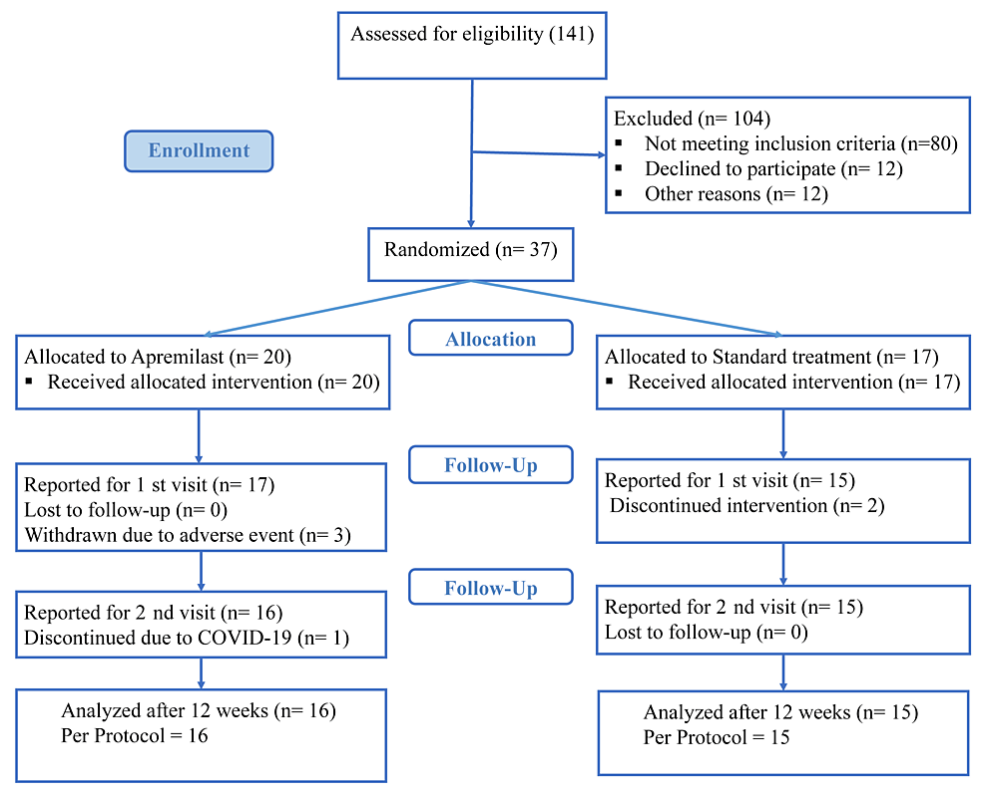
CONSORT flow diagram of participants enrolled in the study

Overall, 141 vitiligo patients were screened for the unstable and non-segmental nature of vitiligo, of which 37 patients, including 21 men and 16 women, were enrolled in the study (Figure 1). The study participants were randomized into two groups; the control group received standard-of-care treatment as prescribed by the dermatologist. The add-on apremilast group accepted standard of care plus “add-on” apremilast. Among different combinations of standard therapy, topical steroids and tacrolimus were both groups' most commonly used drugs. The demographic description of the population is summarized in Table 1.

**Table 1:** Baseline characteristics of participants in Standard treatment and add-on apremilast groups

Demographic Characteristics	Standard treatment (n=17)	Standard treatment and add-on apremilast (n=20)	p-value
Age (Years) Mean (SD)	33.71 (13.1)	31.7 (11.17)	0.619
Gender (male/female)	10/7	11/9	0.815
Height (cm.) Mean (SD)	164.47 (12.80)	164.35 (8.43)	0.974
Weight (kg.) Mean (SD)	67.81 (16.52)	67.3 (12.75)	0.916
BMI Mean (SD)	24.85 (4.46)	24.89 (4.07)	0.977
Clinical Examination (within normal limit)	17 (100%)	20 (100%)	0.243
Disease Duration Median (IQR)	3 (1.5-7)	4 (2-7.75)	0.56
NB-UVB	2	2	N/A
Steroids (Oral)	4	8	N/A
Steroids (Topical)	17	19	N/A
Tab. Azathioprine	0	1	N/A
Tacrolimus (Topical)	15	17	N/A
Melanocyl lotion	2	2	N/A
Sper lotion	1	2	N/A
Tab. Patchex	2	1	N/A
Antioxidants	6	2	N/A
Tab. Calcium	2	6	N/A

Overall, the halt in progression was seen in 93.75% of patients in the add-on apremilast group compared to 66.66% of patients in the control group. However, on fisher’s exact test, the difference was not statistically significant between groups (p=0.083). Repigmentation was seen in 87.50% and 66.66% of the add-on apremilast and control groups, respectively. The median (interquartile range) timeline to observe the first sign of re-pigmentation was 1 (0.87-1.12) month in the add-on apremilast group and 1.75 (1-2) month in the control group, suggesting significantly earlier re-pigmentation in add-on apremilast group (p=0.018) in Table 2.

**Table 2:** Treatment response in standard treatment and apremilast treatment groups after the study period ^#^Inter group comparison was performed using Fisher’s Exact Test and ^$^Mann-Whitney Test, P-value less than 0.05 was considered as significant.

Serial number	Response to treatment	Standard treatment (n=15)	Standard treatment + add-on apremilast (n=16)	p-value
1	Halt in progression	10/15 (66.66%)	15/16 (93.75%)	0.08^#^
2	Re-pigmentation	10/15 (66.66%)	14/16 (87.5%)	0.22^#^
3	1^st^ Sign of Repigmentation (months) Median (IQR)	1.75 (1-2)	1 (0.87-1.12)	0.018^#,$^

**Figure 2:**
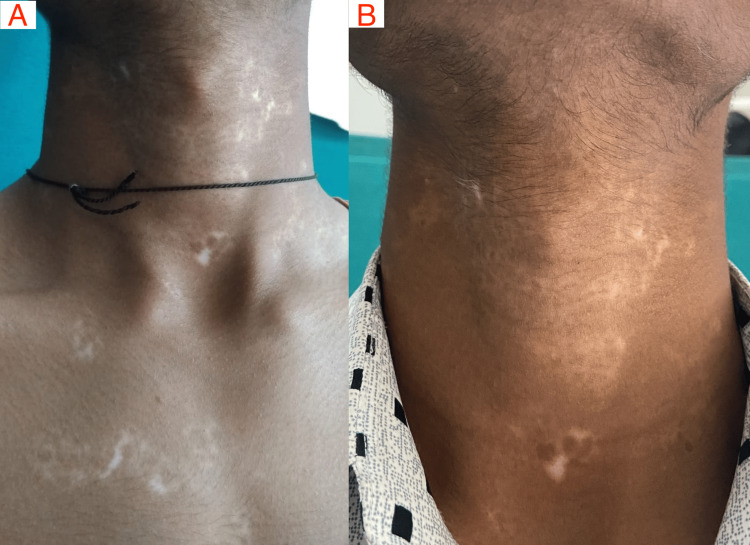
Response to the treatment in standard treatment control group: (A) Baseline and (B) after 12 weeks of treatment

**Figure 3:**
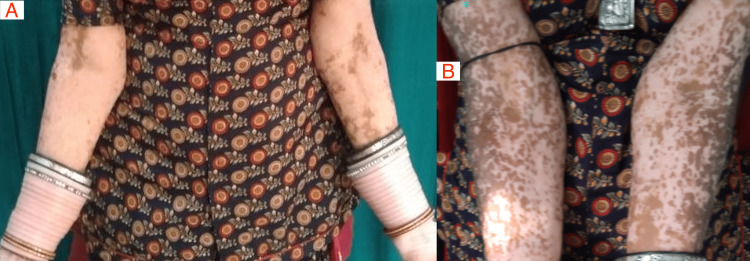
Response to the treatment in the add-on apremilast group: (A) Baseline and (B) after 12 weeks of treatment

Over 12 weeks of study duration, the median BSA score reduced significantly (p= 0.023) in the add-on apremilast group from 12.50 (2.37-77.5) to 10.75 (4-73.7). However, the median BSA score in the control group was the same (5.0) in both pre and post-treatment (p=1.00). The median VASI score also reduced significantly in the add-on apremilast group from 7.45 (0.9-39.9) to 6.21 (1.4-35.3) (p= 0.010) while it non-significantly decreased in the control group, i.e., from 1.45 (0.4-5.8) to 1.4 (0.52-4.87) (p= 0.754). In both the groups, there was a significant decrease in DLQI score, i.e., from 4.40±3.81 to 4.33±3.33 (p=0.023); also, in the add-on apremilast group, there was a statistically significant (p=0.003) difference of 2.88±3.66 in DLQI score from baseline, i.e., from 6.88±5.03 to 4.00±3.22 was recorded. There is an increase of 8.67(±15.17) in the VAS score in the control group at 12 weeks, i.e., from 33.00 (±18.10) to 41.67 (±25.61) (p=0.044). In the add-on apremilast group, there was a statistically significant (p<0.001) difference in VAS score over 12 weeks, i.e., from 44.69 (±17.65) to 64.69 (±21.71), with an improvement of 20 (±16.63) in the group. There was a non-significant alteration in the Body Mass Index (BMI) in the control group from 24.93±4.60 to 25.04±4.42 (p>0.05), whereas, in the add-on apremilast group, there was a statistically significant (p=0.003) decrease in BMI score from 25.09±4.43 to 24.47±4.26; a reduction of 0.62±0.65 kg/m^2^ was recorded (Table 3). The clinical appearance of the affected organ at baseline and after treatment duration is represented in Figures 2, 3 in the standard treatment and apremilast groups, respectively.

**Table 3:** Treatment response in standard treatment and add-on apremilast groups over the period BSA = Body Surface Area, VASI =Vitiligo Area and Severity Index score, DLQI = Dermatology Life Quality Index, VAS = Visual Analogue Scale ^#^ Intra group comparison was performed using Paired-t test, *P-value less than 0.05 was considered as significant.

Parameters	Treatment groups /variables	Baseline	12 weeks	p-value^#^
BSA Median (IQR)	Standard treatment (n=15)	5 (2,10)	5 (1.5, 12)	1.000
	Standard treatment + add-on apremilast (n=16)	12.50 (2.37, 77.5)	10.75 (4, 73.75)	0.023*
VASI Median (IQR)	Standard treatment (n=15)	1.45 (0.4, 5.8)	1.40 (0.52, 4.87)	0.754
	Standard treatment + add-on apremilast (n=16)	7.45 (0.91, 39.98)	6.21 (1.41, 35.37)	0.010*
DLQI Mean (± SD)	Standard treatment (n=15)	4.40 (± 3.81)	4.33 (± 3.33)	0.023*
	Standard treatment + add-on apremilast (n=16)	6.88 (± 5.03)	4.00 (± 3.22)	0.003*
VAS Mean (± SD)	Standard treatment (n=15)	33.00 (± 18.1)	41.67 (± 25.61)	0.044*
	Standard treatment + add-on apremilast (n=16)	44.69 (± 17.65)	64.69 (± 21.71)	0.001*
BMI Mean (± SD)	Standard treatment (n=15)	24.93 (± 4.6)	25.04 (± 4.42)	0.056
	Standard treatment + add-on apremilast (n=16)	25.09 (± 4.43)	24.47 (± 4.26)	0.003*

Forty-five adverse events in 16 patients were observed in the add-on apremilast group. Weight loss, headache, and diarrhea were the most frequent adverse events. The adverse events were predominantly seen within the first fifteen days of initiating the drug and subsequently subsided within 2-3 days without any treatment. One patient reported depression and undertook psychiatric consultation in the add-on apremilast group. Eight adverse events in seven patients were observed in the control group. No serious adverse events were observed in this group. On comparing adverse events between both the groups, only diarrhea and weight loss came out significantly more in the add-on apremilast group (p=0.024 and p=0.002, respectively) (Table 4). 

**Table 4:** Adverse events in standard treatment and add-on apremilast groups ^#^Inter-group comparison was performed using Fisher’s Exact Test, *p-value less than 0.05 was considered significant.

S. No.	Adverse Drug Event	Standard treatment (n=17)	Standard treatment + add-on apremilast (n=20)	p-value^#^
1	Diarrhoea	0	6	0.024*
2	Nausea	0	4	0.093
3	Upper Abdominal Pain	1	3	0.419
4	Appetite loss	0	4	0.093
5	Weight loss	0	9	0.002*
6	Headache	1	7	0.055
7	Insomnia	0	3	0.174
8	Depression	0	1	0.571
9	Fatigue	1	4	0.272
10	URTI	5	4	0.306

## Discussion

The management of vitiligo remains challenging because of its complex pathogenesis. It involves a personalized approach, and the treatment choice depends on factors like disease duration, distribution, activity, impact, skin type, extent, sex, age, involved areas, social life, and cultural influences. Due to different clinical presentations and distinct underlying pathogenetic mechanisms, non-segmental vitiligo (NSV) and segmental vitiligo (SV) have different treatment paths. Apremilast for vitiligo was first tried in 2017 because of its anti-inflammatory property in a case of resistant vitiligo in a 52-year-old woman. The patient experienced fairly significant re-pigmentation and reported around 70% improvement on 11 months of treatment with apremilast at 30mg twice daily and two shots of 60mg triamcinolone, first at three months a repeat shot at 4.5 months of treatment [16]. Another case of a 59-year-old man with vitiligo and psoriasis reported clearance of almost all psoriatic lesions and re-pigmentation of the hypopigmented patches on the neck and lower face after six months of treatment with apremilast [17].

The response to treatment was recorded as a halt in the progression and re-pigmentation of lesions. The break in progression was seen in 93.75% of patients in the add-on apremilast group compared to 66.66% in the control group. However, the change from baseline was not statistically significant between groups. The re-pigmentation difference was also nonsignificant between both groups, i.e., 87.5% and 66.66% in the add-on apremilast and control group, respectively. While Majid et al. have reported 100% stabilization of disease and 61.5% (eight out of 13) cases showing improvement (partial re-pigmentation) with three months of apremilast and topical tacrolimus 0.1% [18]. The better outcome in our group regarding re-pigmentation could be because of the difference in other standard drugs being allowed in our study. At the same time, it was limited to topical tacrolimus only in case series [18].

Apremilast, approved for psoriasis and psoriatic arthritis, has been shown to regulate inflammatory mediators and might be a promising therapeutic option [19]. It is a PDE4 inhibitor that increases the level of intracellular cAMP by inhibiting the degradation of cAMP by the phosphodiesterase enzyme [20]. This results in the alteration of many pro- and anti-inflammatory mediators like TNFα, IL -2, IL-5, IL- 6, IL- 8, IL-10, IL-12, IL-13, IL-17, IL-23, and IFN-γ [21,22]. It could also impact melanocyte growth and differentiation by increasing the levels of cAMP [22]. Therefore, with its proven efficacy and safety in a few studies earlier, we here attempt to dig further in the search for its safety and efficacy in vitiligo.

The median BSA score reduced significantly from 12.50 (2.37-77.5) to 10.75 (4-73.7) with p=0.023 in the add-on apremilast group, but it remained the same over 12 weeks in the control group (p >0.99). Similarly, Kim et al. reported a significant reduction in BSA (p=0.0001) at the end of 16 weeks of combination therapy of apremilast and Narrow-band ultraviolet B (NB- UVB) phototherapy group than NB-UVB monotherapy in skin types IV-VI [23]. This could be due to apremilast having some potentiation effect on NB-UVB phototherapy and may expedite re-pigmentation of photo-type IV-VI.

Vitiligo Area Scoring Index (VASI) is considered a quantitative parametric score to compare the efficacy of different treatment modalities in vitiligo. In our study, at the end of 12 weeks of treatment, the median (IQR) VASI score decreased significantly (p=0.010) from 7.45 (0.9-39.9) to 6.21 (1.4-35.3) in add-on apremilast group while the reduction was not significant in the control group (p=0.754) from 1.45 (0.4-5.8) to 1.4 (0.52-4.87). Khemis et al. also reported a decrease in VASI score from 12.90 (5.70±39.60) to 10.00 (5.50±24.30) after 24 weeks of treatment with apremilast plus ultraviolet B (UVB) group [24]. Another split-body pilot study by Kim et al., involving 14 vitiligo patients reported a greater reduction in mean VASI score with combined therapy of apremilast and NB-UVB phototherapy than NB-UVB monotherapy at the end of 16 weeks (p=0.0001) [23]. Similarly, Majid et al. reported a 7.11% mean reduction in VASI among the responders [18]. Thus, the findings in VASI are similar to other studies. The DLQI score was also significantly decreased in our study in the add-on apremilast group over 12 weeks of treatment (mean difference -2.88, p=0.003). We evaluated patient satisfaction through the VAS scale, which increased significantly in both add-on apremilast groups (p=0.001) and the control group (p=0.044). Majid et al. reported a mean patient satisfaction score of 6.07 on the 1-10-point scale at the end of the treatment in a case series of 13 patients [18]. Similarly, our study reported a mean patient satisfaction score of 64.7 on the 1-00-point scale in the add-on apremilast group at 12 weeks. Thus, the findings for patient satisfaction are similar to other studies. Khemis et al. said the median time for the first sign of re-pigmentation was 43.5 days in the apremilast group plus UVB group and 65 days in the placebo plus UVB group (p=0.105) [24]. In our study mean time to observe the first sign of re-pigmentation was 1.0 months (30 days) in the add-on apremilast group and 1.75 months (52 days) in the control group. This difference might be due to the variation in other standard treatments (topical steroids, oral steroids, azathioprine, tacrolimus, etc.) available to our study participants. In contrast, it was restricted to only UVB or placebo. In our study, BMI slightly increased in the control group while decreasing in the add-on apremilast group (p=0.003). Van Voorhees et al. reported more reduction in BMI in the apremilast group compared to the placebo group [25].

Forty-five adverse events in 16 patients were observed in the add-on apremilast group. Weight loss, headache, and diarrhea were the most frequent adverse effects. The adverse events were predominantly seen within the first fifteen days of initiating the drug and subsequently subsided within 2-3 days without any treatment. One patient reported depression and took psychiatric consultation in the add-on apremilast group. Eight adverse events in seven patients were observed in the control group. However, no serious adverse events were observed in the control group. On comparing adverse events between both the groups, only diarrhea and weight loss came out significantly more in the add-on apremilast group (p=0.024 and p=0.002, respectively).

This study had a few limitations. This study was conducted at a single center with a small number of patients owing to the COVID-19 pandemic. Pictures were the primary source of data collection during follow-up, given patients’ inability to attend the outpatient department. However, some patients were uncomfortable sending pictures, and their scores were calculated through video calling. The quality of pictures depended on the camera, resulting in a lesser optimal assessment of disease progression and subjective differences in evaluation. The small sample size, similar demography, and shorter study duration added to the limitations. The validity and applicability of the trial findings are limited due to the abovementioned reasons. Further studies with adequate sample size and outpatient follow-up instead of telephonic and photographic ones might lessen the subjective errors in calculating scores and improve the outcome.

## Conclusions

In conclusion, the onset of observable remission (halt in progression & re-pigmentation) was better in the add-on apremilast group. However, the results were comparable. VASI, BSA, DLQI, and BMI scores were significantly reduced in the add-on apremilast group over week-12. However, the results were similar. Treatment with add-on apremilast accelerated clinical improvement. It also reduced disease progression and improved the disease index among participants. However, add-on apremilast had a lower tolerability profile than the control group.
